# Supplementary material for: Integrative proteomic profiling of ovarian cancer cell lines reveals precursor cell associated proteins and functional status
Source: Nat Commun. 2016 Aug 26;7:12645. doi: 10.1038/ncomms12645 (PMC5007461; doi:10.1038/ncomms12645)
Supplement: Supplementary Information — Supplementary Figures 1-6 and Supplementary Table 1 [file ncomms12645-s1.pdf]

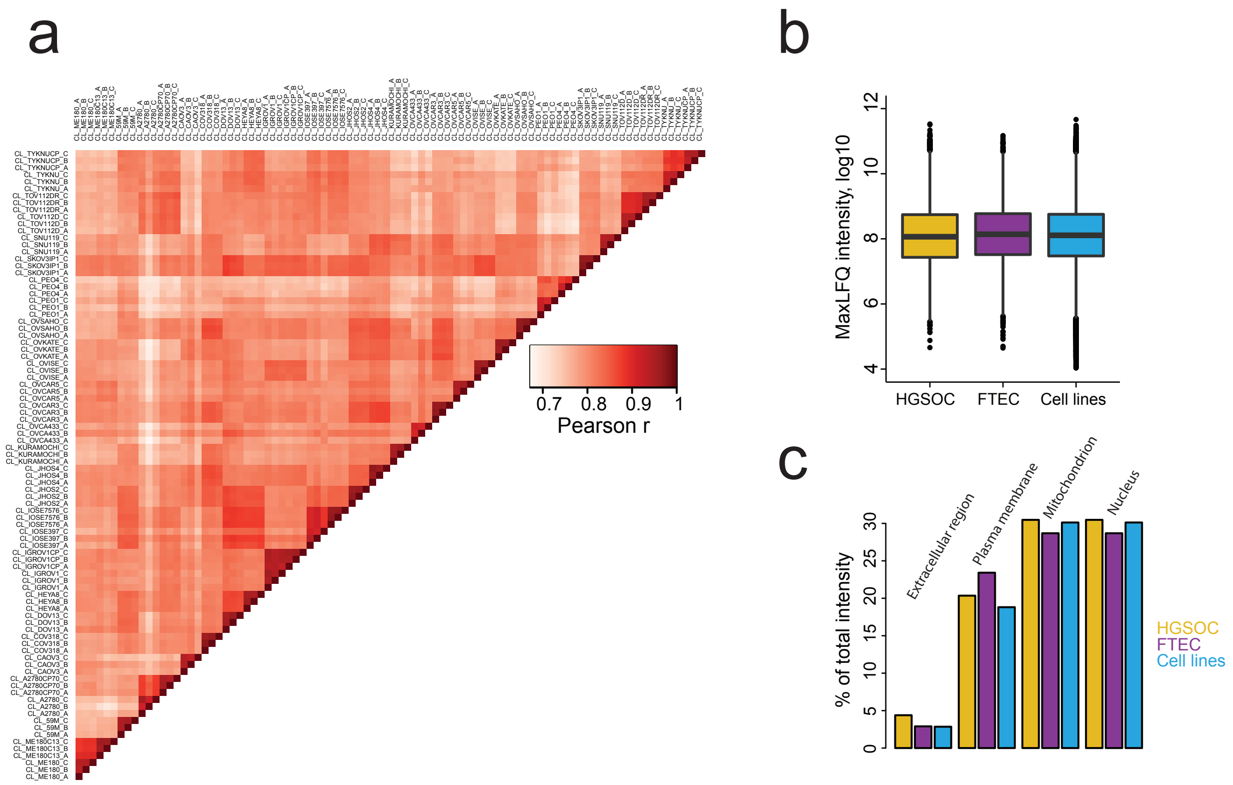

Supplementary Figure 1: Proteomics of 30 cell lines

(a) Pearson correlations of cell line replicates. To evaluate the reproducibility of the label-free quantification, a correlation matrix was generated for each pairwise comparison based on Pearson correlation values.

(b) Protein intensity distribution across all sample types. Protein intensity distributions in tumor tissues, cell lines and primary cells were compared. Protein intensities of all three systems are equally distributed over a range of more than six orders of magnitude indicating a very similar dynamic range of protein expression in the HGSOC tumors, FTECs and cell lines.

(c) Proportional protein levels of selected Gene Ontology Cellular Compartment (GOCC) pathways. Gene ontology annotations were applied to the data and total summed intensities for each depicted pathway were calculated relative to the total protein intensity in each of the three systems. The bar chart shows that there is comparable proportional protein expression in the GOCC pathways “Extracellular region”, “Mitochondrion”, “Nucleus”, and “Plasma membrane” between the cell lines, HGSOC tumors, and FTECs.

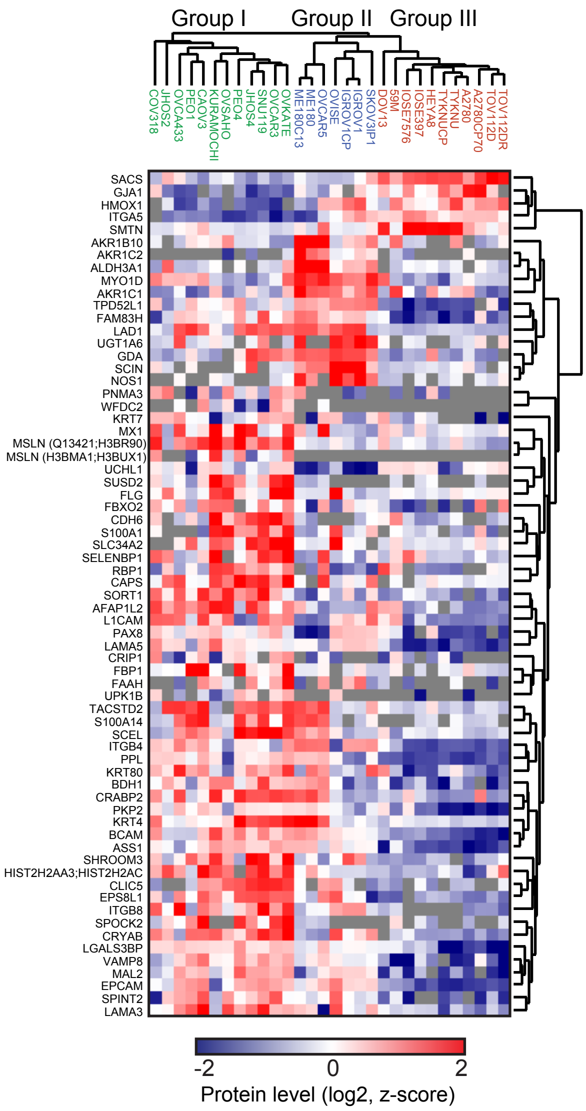

Supplementary Figure 2. A 67-protein signature distinguishes between the three cell lines groups. Proteins that strongly discriminated between the three main cell line groups were selected using feature ranking based on p-values computed from modified test statistic and Support Vector Machines classifications. Protein levels were normalized by z-scoring and clustered using Spearman's rank correlation as a distance measure. The selected 67 top ranked proteins, distinguishing each cell line group from the other two, form cell line group-specific clusters of upregulated (in red) and downregulated (in blue) proteins. Grey values indicate undetected proteins.

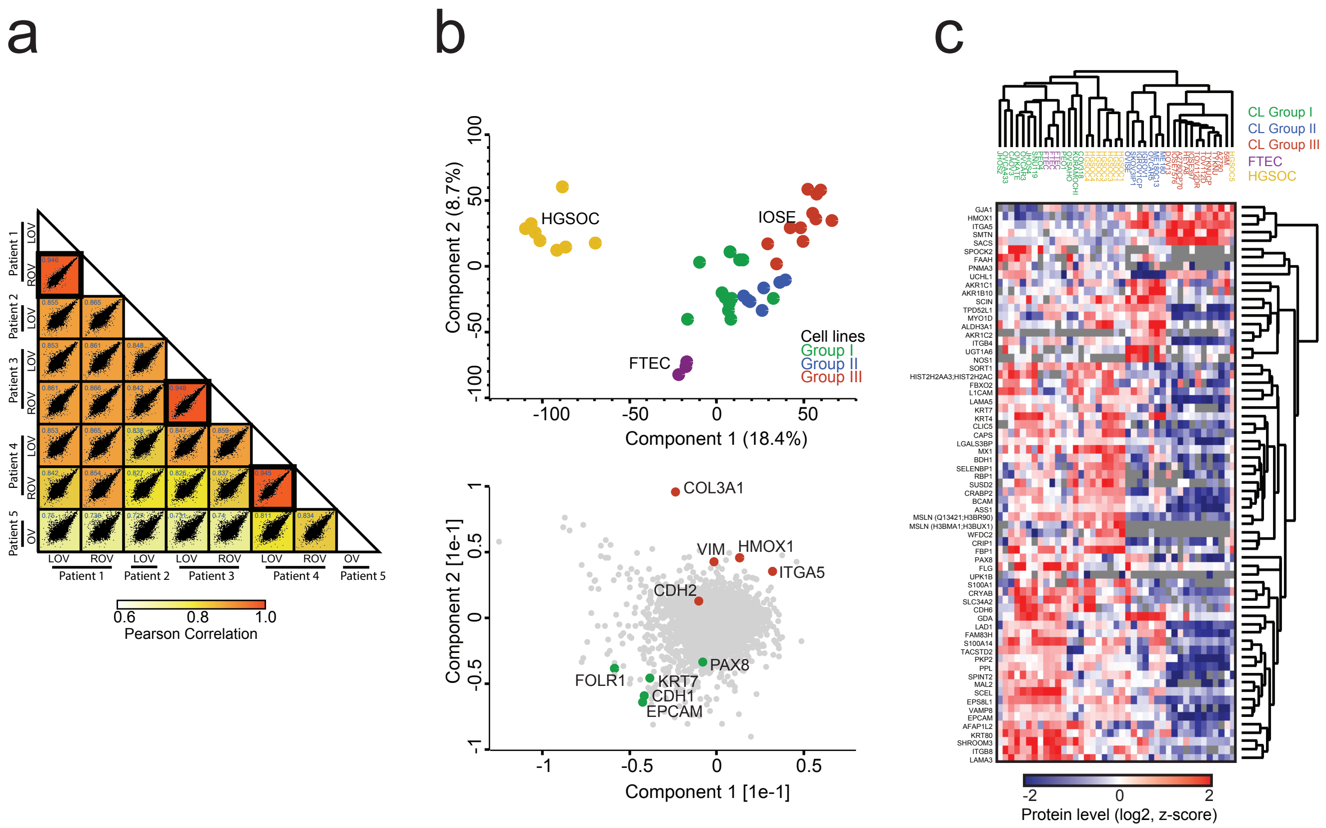

Supplementary Figure 3. Proteomic correlation of HGSOC tissues and clustering with cell lines and primary cells.

(a) Pearson correlations of the HGSOC tumors. To evaluate the reproducibility of the label-free quantification in the HGSOC ovarian tumors, Pearson correlations were calculated between bilateral tumors from the same patient (n = 3, mean Pearson correlation = 0.95) and between tumors from different patients (Pearson correlation = 0.72-0.87).

(b) Proteins driving the PCA separation in Fig. 4b. The driver proteins of component 2 included the known epithelial proteins EPCAM, CDH1, PAX8, KRT7, and FOLR1 (green) as well as the mesenchymal proteins COL3A1, VIM, ITGA5, HMOX1, and CDH2 (red).

(c) Unsupervised hierarchical clustering analysis of all samples based on the 67-protein signature. Z-scored protein levels were used to cluster all samples based on Spearman rank correlations for column and row-wise clustering. The dendrogram shows that the samples fall into two core clusters based on this signature. Values shown in grey were not detected.

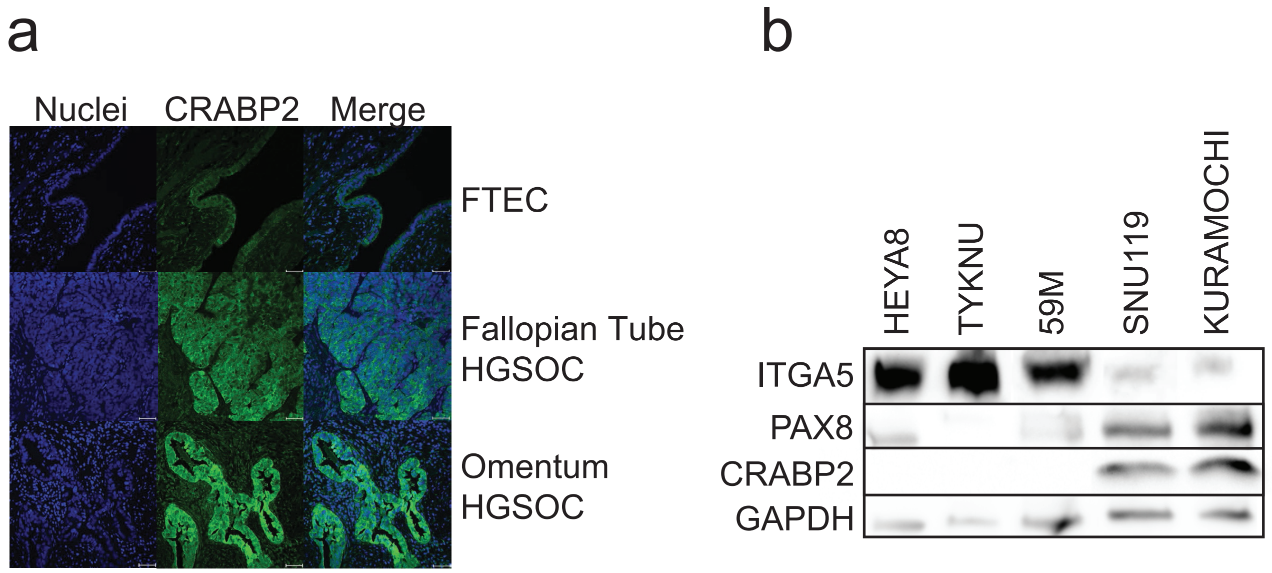

Supplementary Figure 4. Orthogonal validation of CRABP2, ITGA5, and PAX8 expression by immunofluorescence or Western Blot.

(a) Immunofluorescence staining for CRABP2 in formalin-fixed paraffin-embedded (FFPE) sections of normal FTECs, fallopian tube HGSOC, and omentum HGSOC. FFPE sections were stained with an anti-CRABP2 antibody and detected with an Alexa Fluor 488-labelled antibody.

(b) Western Blot for CRABP2, PAX8, and ITGA5 in cell lines. Cell line lysates from group I (SNU119, KURAMOCHI) and group III (HEYA8, TYKNU, 59M) cell lines were collected in RIPA buffer and 20 µg protein from each was electrophoresed on 4-20% resolving gels. Following transfer, membranes were blotted with antibodies against ITGA5, PAX8, CRABP2, and GAPDH, followed by HRP-linked secondary antibodies. Protein bands were visualized on the Syngene G:BOX gel imaging system.

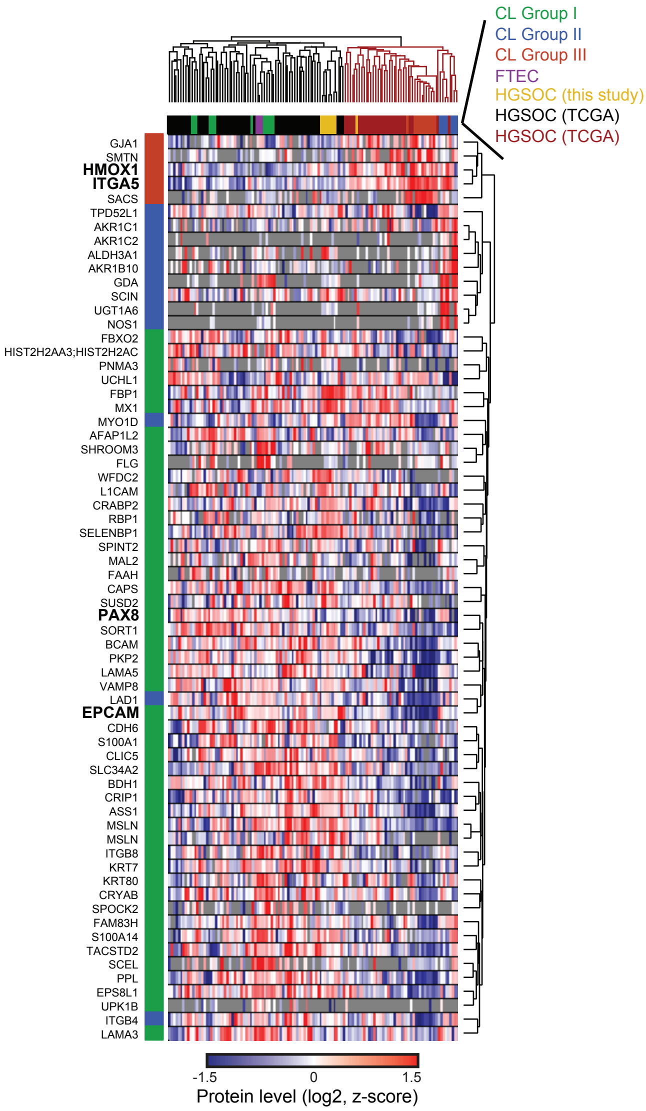

Supplementary Figure 5. Clustering of the TCGA tumors based on the 67-protein cell line signature.

Hierarchical clustering of 84 TCGA tumors with the cell lines, FTECs, IOSEs, and HGSOC-1 to -5. Hierarchical clustering based on the 67-protein signature was applied to the publically available proteomic profiles of 84 TCGA tumors and our own dataset, comprised of cell lines, FTECs, IOSEs, and HGSOC-1 to -5. Z-scoring was performed group-wise for ITRAQ data (TCGA/CPTAC) and our own label-free dataset. Green, blue, and red colors along the left side of the heatmap represent proteins belonging to cell line groups I, II, and III, respectively. Group I proteins, PAX8 and EPCAM, and group III proteins, HMOX1 and ITGA5, are highlighted.

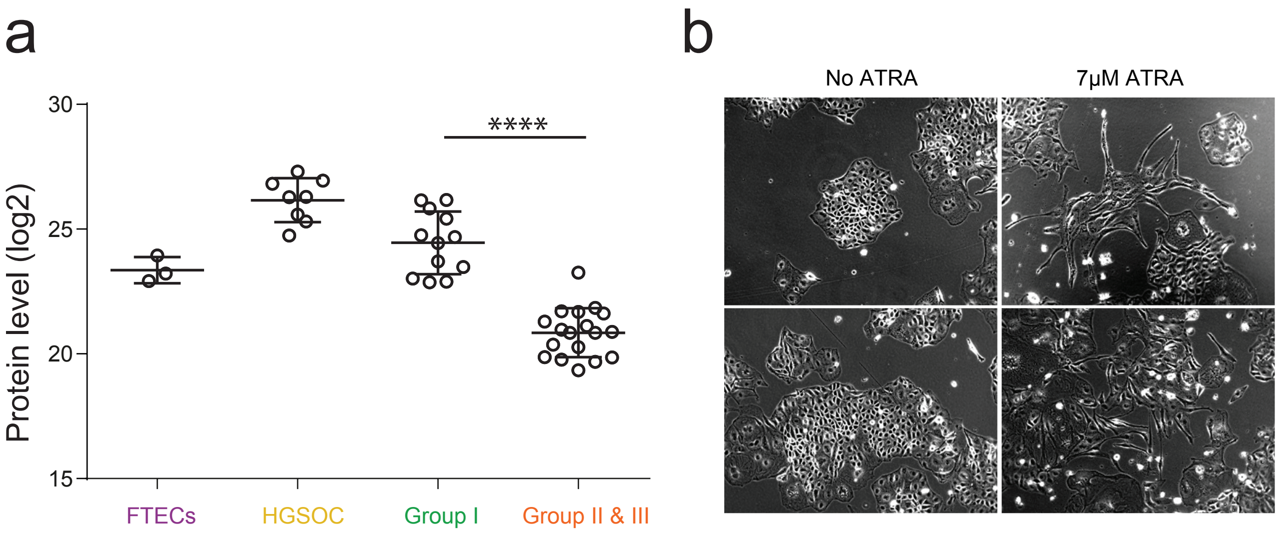

Supplementary Figure 6. Differential expression and regulation of the vitamin A pathway in three cell line groups.

(a) Average LFQ expression of the five quantified vitamin A pathway proteins in the FTECs, HGSOC tumors, group I cell lines, and group II & III cell lines. The average LFQ values for CRABP1, CRABP2, RBP1, RBP4, and RPB7 were calculated and plotted. Average expression of all five proteins was significantly higher in the FTECs, HGSOC tumors, and group I cell lines than in group II & III cell lines. **** < 0.0001

(b) ATRA treatment of the group I cell line KURAMOCHI**.** The KURAMOCHI cell line was treated with 7 μM ATRA daily for 7 days. On Day 8, cells were visualized and imaged. ATRA treatment induced a morphological, but not proliferative (Fig. 6d), change in the cells.

Supplementary Table 1. Example driver proteins in cell line Groups I, II and III

Gene Symbol	Protein	UniProt ID	Related Literature on Protein Function and Potential Role in Cancer	References
**Group I**				
CAPS	Calcyphosin	Q13938	Predictive of tamoxifen response in ER+ breast cancer	Johansson et al., 2015
CRABP2	Cellular retinoic acid-binding protein 2	P29373	Mediates the anti-oncogenic effect of all-*trans* RA	Takase et al., 1986; Schug et al., 2007; Schug et al., 2008
CRYAB	αB-crystallin	P02511	Inhibits TRAIL-and cisplatin-induced apoptosis in OvCa cells	Volkmann et al., 2013
KRT7	Keratin, type II cytoskeletal 7	P08729	Epithelial cell marker used in differential diagnosis	Berezowski et al., 1996 Lengyel, 2010
MSLN	Mesothelin	Q13421	Marker for detecting ovarian epithelial cancers<br>Inhibits chemotherapy-induced apoptosis in OvCa<br>Promotes migration and invasion of OvCa cells	Huang et al., 2006;<br>Chang et al., 2009;<br>Chang et al., 2012
**Group II**				
AKR1C1	Aldo-keto reductase family 1 member C1	Q04828	Higher in CCC OvCa than in other subtypes<br>Expressed in normal kidneys and in renal carcinomas of clear cell origin<br>Induced with the development of cisplatin resistance in OvCa and other cancers<br>Detoxifies intracellular ROS in cisplatin-resistant cell lines	Schaner et al., 2003;<br>O' Conner et al., 1999;<br>Matsunaga et al., 2013;<br>Chen et al., 2005;<br>Chen et al., 2008
AKR1C2	Aldo-keto reductase family 1 member C2	P52895	Induction of AKR1C2 contributes to cisplatin resistance in bladder cancer cells through antioxidative effects	Shirato et al., 2014
AKR1B10	Aldo-keto reductase family 1 member B10	O60218	Decreases RA synthesis and depletes RA transactivation in cell lines, which may lead to dedifferentiation and tumorigenesis	Ruiz et al., 2009
NOS1	Nitric oxide synthase, brain	P29475	NOS1/NOS3 contributes to chemoresistance by suppressing cisplatin-induced apoptosis in isogenic cisplatin-resistance OvCa cell lines in a p53-independent manner	Leung et al., 2008
SCIN	Adseverin	Q9Y6U3	Marker of cisplatin-resistance in bladder cancer cell line<br>Knockdown of SCIN inhibits human prostate cancer cell proliferation	Miura et al., 2012<br>Wang et al., 2014
UGT1A6	UDP-glucuronosyltransferase 1-6	P19224	UGT1A6 polymorphisms are associated with lung, breast, and colorectal cancer risk<br>UGT1A6 promoter is regulated by the clear cell OvCa marker HNF1B	Auyeung et al., 2003<br>Kua et al., 2012<br>Justernhoven et al., 2013<br>Osawa et al., 2012
**Group III**				
GJA1	Gap junction alpha-1 protein	P17302	Facilitates cell adhesion, invasion, and metastasis in malignant glioma, and breast, prostate, and gastric cancer	Lin et al., 2002; Elzarrad et al., 2008; Kapoor et al., 2004; Lamiche et al., 2012; Tang et al., 2013.
HMOX1	Heme oxygenase 1	P09601	Maintains cellular homeostasis and plays a key role in the adaptive response to cellular stress<br>Contributes to cisplatin-resistance in lung cancer and OvCa cell lines<br>Involved in invasion and metastasis	Stocker et al., 1987;<br>Brouard et al., 2000;<br>Kim et al., 2008; Bao et al., 2014; Song et al., 2009<br>Was et al., 2006; Sunamura et al., 2003; Lee et al., 2008
ITGA5	Integrin alpha-5	P08648	Mediates early OvCa metastasis	Kenny et al., 2014
SACS	Sacsin	Q9NZJ4	Regulator of the Hsp70 chaperone machinery	Parfitt et al., 2009
SMTN	Smoothelin	P53814	Cytoskeletal smooth muscle-specific protein<br>Marker for mesenchymal tumours of the digestive tract	Kramer et al., 1999;<br>Coco et al., 2009
